# Phage therapy as a revolutionary medicine against Gram-positive bacterial infections

**DOI:** 10.1186/s43088-021-00141-8

**Published:** 2021-08-28

**Authors:** Archana Loganathan, Prasanth Manohar, Kandasamy Eniyan, C. S. VinodKumar, Sebastian Leptihn, Ramesh Nachimuthu

**Affiliations:** 1 School of Bioscience and Technology, Vellore Institute of Technology (VIT), Vellore, Tamil Nadu India; 2 Zhejiang University-University of Edinburgh (ZJU-UoE) Institute, Zhejiang University, School of Medicine, Haining, 314400 Zhejiang People’s Republic of China; 3 School of Medicine, The Second Affiliated Hospital Zhejiang University (SAHZU), Hangzhou, Zhejiang People’s Republic of China; 4 Department of Microbiology, S.S. Institute of Medical Sciences and Research Centre, Davanagere, India; 5 Department of Infectious Diseases, Sir Run Run Shaw Hospital, Zhejiang University School of Medicine, Hangzhou, People’s Republic of China; 6 Infection Medicine, Biomedical Sciences, Edinburgh Medical School, College of Medicine and Veterinary Medicine, The University of Edinburgh, 1 George Square, Edinburgh, EH8 9JZ UK

**Keywords:** Phage therapy, Antibiotic resistance, Phage commercialization, Phage products, Future medicine, Gram-positive pathogens

## Abstract

**Background:**

Antibiotic resistance among pathogenic bacteria has created a global emergency, prompting the hunt for an alternative cure. Bacteriophages were discovered over a century ago and have proven to be a successful replacement during antibiotic treatment failure. This review discusses on the scientific investigation of phage therapy for Gram-positive pathogens and general outlook of phage therapy clinical trials and commercialization.

**Main body of the abstract:**

This review aimed to highlight the phage therapy in Gram-positive bacteria and the need for phage therapy in the future. Phage therapy to treat Gram-positive bacterial infections is in use for a very long time. However, limited review on the phage efficacy in Gram-positive bacteria exists. The natural efficiency and potency of bacteriophages against bacterial strains have been advantageous amidst the other non-antibiotic agents. The use of phages to treat oral biofilm, skin infection, and recurrent infections caused by Gram-positive bacteria has emerged as a predominant research area in recent years. In addition, the upsurge in research in the area of phage therapy for spore-forming Gram-positive bacteria has added a wealth of information to phage therapy.

**Short conclusion:**

We conclude that the need of phage as an alternative treatment is obvious in future. However, phage therapy can be used as reserve treatment. This review focuses on the potential use of phage therapy in treating Gram-positive bacterial infections, as well as their therapeutic aspects. Furthermore, we discussed the difficulties in commercializing phage drugs and their problems as a breakthrough medicine.

## Background

The rising antibiotic resistance is troubling the healthcare system, encouraging researchers to look at new or modified antibiotics. However, discovering a new antibiotic is a daunting task that may take several years, and the possibility of bacteria showing resistance to newly found antibiotics will always remain unanswered [111]. Bacterial mechanisms are so fast that resistance to antibiotics develops in a shorter period. Notably, a few large pharmaceutical firms, including Novartis and Sanofi, have ceased antibiotic research and development [73, 101]. Consequently, there is an increasing need for the global scientific community to look for new therapeutics focused on non-antibiotic therapies to meet the challenge. Phage therapy is one of the oldest and effective therapies and is recommended as a superior medication in a few European countries and Russia (the former Soviet Union). The bacteriophage is a ubiquitous virus that preys on bacteria (host) for their multiplication and kills them at the end of the process, a tool for releasing progeny particles [99, 110]. Ernest Hankin, a British bacteriologist, identified the first antibacterial agents from the Ganges in 1896, but he could not name the agent involved in bacterial killing. Later in 1915, Fredrick Twort speculated that the agents were viruses. It was Felix d’Herelle in 1917 who called it a ‘bacteriophage’ and conducted his first human test on a patient at Paris' Enfants Malades hospital. Based on their early success, bacteriophages were used to treat bacterial infections and continue to be an effective tool in Georgia, Poland, and Russia. However, much of the Western world refused to consider phage therapy due to a lack of clinical trials to prove its effectiveness. Since the discovery of antibiotics in the 1940s, the use of phages in therapy *has lost its importance* in Western medicine. Antibiotics have taken many forms since their discovery to be an effective antibacterial medicine. However, constant antibiotic pressure has resulted in antibiotic-resistant development in bacteria, which ultimately contributes to the post-antibiotic era, which began in the late 2000s. Treatment failure and the number of deaths caused by antibiotic-resistant bacterial infections are increasing linearly. This situation has recalled the importance of bacteriophages, and now, there is a renewed interest in phage therapy.

Bacteriophages (in medicine) are well known for their properties such as (a) self-replication, which reduces the need for several doses during treatment; (b) target specificity, which renders normal flora harmless; (c) they do not interact with mammalian cells; (d) they have a high ability to cross the blood–brain barrier, and (e) their ability to disrupt bacterial biofilms [41]. All of this importance demonstrates that phages have a distinct mode of action than chemotherapeutic agents, making them an effective antibacterial agent [52]. Lytic bacteriophages are used in therapy because they destroy bacteria and reduce bacterial load in a short period. *On the other hand*, the lysogenic phage incorporates its genome into its host chromosomal DNA and replicates, therefore, carrying circulating toxin or other virulence genes from the previous cycle. So lysogenic phages can mobilize virulence or resistance genes, rendering them unsuitable for therapy [47, 76]. Well-developed and flexible genetic pathways in Gram-positive bacteria appear to exhibit susceptibility to most chemical drugs. World Health Organization (WHO) published a list of global priority pathogens based on global infections and their importance in public health. Among them, three Gram-positive bacteria, *Staphylococcus aureus*, *Enterococcus faecium*, and *Streptococcus pneumoniae*, were prioritized as high and medium pathogens. Notably, these bacteria are becoming resistant to the last-resort antibiotics making treatment complicated [4]. Most Gram-positive bacteria link to healthcare and community-associated diseases, notably *S. aureus* and *S. pneumoniae* [14, 105]. Gram-positive bacteria have adapted to respond to the selective pressure in the antibiotic system, resulting in the proliferation of antibiotic-resistant and, ultimately, resistance transmission to other co-colonized bacterial species. For instance, in *S. pneumoniae*, antibiotic stress has resulted in gene regulon transcription, resulting in an increasing exchange of resistance genes in the co-colonized bacterial population [28, 76].

Phage research against Gram-positive bacteria began before antibiotics discovery. Two age-old companies, Eli Lilly and Eliava phage therapy center, are known for their large repository of bacteriophages against Gram-positive bacteria. The therapeutic bacteriophages against Gram-positive bacteria fall under the order—*Caudovirales* that comprise nine families: *Ackermannviridae, Autographiviridae, Chaseviridae, Demerecviridae, Drexlerviridae, Herelleviridae, Myoviridae, Podoviridae,* and *Siphoviridae*. Bacteriophages belonging to the order *Caudovirales* have a double-stranded DNA. Members of the *Caudovirales* have a distinct morphology. *Myoviridae* has a long contractile tail, whereas *Podoviridae* have a short non-contractile tail, and *Siphoviridae has* a long non-contractile tail. [6, 7, 25, 51]. While *Ackermannviridae* and *Herelleviridae* are distinct families, they share similar architecture to *Myoviridae*, except *Ackermannviridae* having a star-like spike at the base of the tail. To date (as of 2nd August 2021), 4847 complete genomes of *Caudovirales* are in the GenBank’s RefSeq database, of which 63 belong to *Ackermannviridae*, 136 *Herelleviridae*, 1018 *Myoviridae*, 720 *Podoviridae*, 2310 *Siphoviridae* family, 387 *Autographiviridae*, 14 *Chaseviridae,* 87 *Demerecviridae,* and 112 *Drexlerviridae* [3, 81].

Several experiments on phage therapy prove that it is an effective antibacterial agent against a wide range of bacterial infections. However, bringing them into reality is the most difficult challenge in the current scenario. Accessibility determines the benefit of any medicinal substance for human use. Therefore, researchers, policymakers, and regulatory authorities can work together to make phage therapy a viable alternative to antibiotics. This review will apprise on the current status of phage therapy, challenges facing phage therapy, commercialization of the phages, and the future of phage therapy.

## Main text

### Scrutinizing phages for therapy

The isolation of bacteriophages against few bacterial species is rather tricky irrespective of their abundance in nature. For example, several researchers have previously reported the difficulty of isolating *Staphylococcus* and *Clostridium* phage, considering their large diversity [64]. For a phage to get accepted for treatment, it requires proper characterization before clinical trials. At present, a therapeutic phage is characterized based on many criteria such as (A) polyvalent nature (broad-host range activity), (B) life cycle parameters (adsorption, latency, and burst size), (C) morphological analysis (using TEM, transmission electron microscope), (D) genome analysis (*w*hole *g*enome *s*equencing), (E) the appearance of bacteriophage insensitive mutants (phage-resistance), and (F) the purity of phages (removal of endotoxin). Gram-positive bacteria that are profoundly infectious belong to the genus *Streptococcus*, *Staphylococcus,* and *Clostridium*, while others are more often involved in causing mild infections. In general, phage characterization for therapy begins with determining the host range and continuous up to clinical trials that give a comprehensive overview of therapeutic efficiency and the clinical outcome [90]. The host range of a phage is an evolutionary process [54, 65]. One of the favorable circumstances of bacteriophage is its specificity which does not disturb the bacterial cells outside their host range and guarantee that normal flora remains flawless. The phage–bacterial interaction is between their receptors. This lock and key mechanism are unique for a phage and a bacterium, it depends on the receptor of the bacteria and the ability of the phage to recognize bacterial receptors. The host range of phages can vary from narrow to broad, but the host range in treatment can vary between the patients based on the infecting bacteria at the infection site. It is preferable to choose a phage that attacks a broad range of pathogenic species. For example, staphylococcal phage ϕ812 was found to have a host range of 95% to *S. aureus* and 43% against other *Staphylococcus* spp. [82].

Current standard and treatment strategies of phage administration in humans differ by patient history, treatment strategies, mode of administration, etc. [70]. The treatment depends on the specificity of the phages to recognize the bacteria to which a patient is infected. Each phage is unique in its mechanism of action and varies upon numerous factors, personalized therapy or targeted therapy is the method of choice in many phage centers [113]. This therapy uses a specific phage from the repository, called phage bank, to treat an infected patient. Now, phage products are available from numerous companies that rely on generalized phage therapy. The clinical outcome depends on the results from the randomized controlled trials [62]. Thus, companies like Adaptive Phage Therapeutical, USA, have developed a strategy to use personalized phages (from phage bank) to determine the host range and prepares the readymade phage cocktail for treatment [26].

Recently, encapsulation of phage is one of the most prominently studied areas in phage therapy research. Since phage viability is critical throughout therapy, phage encapsulation was adapted to keep them viable and stable during treatment [107]. Encapsulation of phage is to guarantee significant stability to phage, thereby increasing the therapeutic efficiency. In one of the earlier studies by Felix d’Herelle, chemical compounds such as dihydroxy aluminum sodium carbonate were used to reduce the effect of gastric juice (stomach) on phages [72]. Recently, microencapsulation has been found significant in maintaining phage persistence at the site of infection [60]. Encapsulation helps in controlled or continuous discharge at the vicinity of infection. For instance, *Clostridium difficile* that causes severe diarrhea in humans was treated successfully with phages, which requires administered phages to pass through various stress and pH environments. Thus, encapsulation of *Clostridium* phage can improve the therapeutic outcome of phage therapy. A study led by Gurinder et al. showed that encapsulated *Clostridium* phage holds significant protection upon continuous exposure to an acidic environment and also holds phages for a prolonged period at the site of infection [19, 33, 60, 108]. Thus, encapsulated phages can be considered for therapy.

Antibiotic resistance has caused additional pressure on patients due to the increasing hospital stay and the medical costs. Though the cost of phage therapy is unclear, it depends largely on the easy availability of phage therapy centers but is predicted to cost lesser than antibiotic therapy. A comparison of Methicillin-Resistant *Staphylococcus aureus* (MRSA)-infected patients treated with antibiotics and phage therapy showed that the cost spent on phage therapy was half the cost of antibiotics [71]. In many countries (including the USA), phage therapy is an alternative treatment option, particularly for antibiotic-resistant infections. With growing recognition, many countries have established phage therapy centers that will significantly reduce treatment costs.

### Is phage therapy a choice in secondary bacterial infection?

Co-infection and secondary bacterial infection during viral infections are the major problems [59, 114]. *S. aureus* (20%) and *S. pneumoniae* (65%) are the most common bacteria that cause co-infection. A study on the H1N1 pandemic by Ashley et al. [29] has shown 60% of the mortality in pediatric patients were due to bacterial co-infection. An experiment conducted in monkeys showed that the co-infection of influenza A virus with *S. pneumoniae* resulted in severe lower respiratory infections and morbidity within 40 h [8, 13, 75].

The recent pandemic caused by SARS-CoV-2 (severe acute respiratory syndrome-coronavirus-2) was no exception; a study in China showed that 50% of the patients who died of coronavirus disease-19 (COVID-19) had a secondary bacterial infection [45, 55]. The increasing resistance exhibited by some bacteria causing co-infections or secondary bacterial infections has made antibiotics insensitive, using phage therapy against the targeted bacteria could serve this purpose [61]. It is essential to eliminate the co-colonizing bacteria as they enhance the binding of the virus to the mucosal surface leading to the spread of infection into the inner visceral parts [79]. In November 2020, the Food and Drug Administration (FDA, USA) approved the extended use of phage therapy as an expanded assessment of investigational new drug (IND) in critically ill COVID patients with secondary bacterial infections. This study was conducted by Adaptive Phage Therapeutics, Inc. in collaboration with the Walter Reed Army Institute of Research. They have begun a clinical trial registered under the National Institutes of Health (NIH) in the USA (NCT04636554) [1]. At present, co-colonized or secondary infection-causing bacteria are eliminated by administrating antibiotics, which might fail when the colonizing bacteria show resistance to the antibiotics. The use of phage therapy can overcome this drawback. Many studies on phage therapy have reported the adequate bacterial clearance of respiratory infection within 24–48 h. [88], which reduces the bacterial load making the disease less complicated. During treatment, bacteriophage can be administered with nebulizers or through inhalants at a controlled rate [10, 18, 63, 103]. In China, a recent study by Wu et al. proved that phages are effective against secondary bacterial infections in which they successfully treated four COVID-19 patients [112]. The issue related to the simultaneous administration of bacteriophage and viral vaccines remains undiscovered. Thus, there is a need for clinical experimentation to implement phage therapy in treating secondary bacterial infections. Another case where secondary bacterial infections are more prominent is in cancer patients, who are more prone to be affected by *S. aureus* [22]. A study conducted by Weber et al. showed the effect of phage therapy against bacterial co-infections in 20 cancer patients who had a previous history of antibiotic treatment failure. The patients had a bacterial infection caused by resistance strains of *S. aureus*, *P aeruginosa*, *K pneumoniae*, *K oxytoca,* and *E. coli*. For systemic infections, host-specific phages were given orally three times a day for 2–9 weeks, and for localized infections, they were applied topically. This study showed that bacterial infection was cleared without any adverse effects in all the patients. However, the impact of phages on cancer cells was not analyzed [109].

### Oral biofilm and recurrent infections

One of the complications of dental treatment is an implant and oral biofilm. The oral microbiome (biofilm) consists of both Gram-positive and Gram-negative bacteria. *Actinomyces* spp*.*, *Lactobacillus* spp*.*, *Streptococcus* spp*.,* and *Enterococcus* spp. are the most common Gram-positives. Oral biofilm treatment could become much easier with the increasing interest in phage therapy. These bacteria appear to form co-aggregates, resulting in biofilm formation [40, 98, 100]. The biofilms are composed of self-secreted exopolysaccharides (EPS), dividing and non-dividing cells. EPS plays a role in providing an outer coat for the biofilm cells, thereby hindering the interaction of antibiotics with the bacteria. Bacteriophages can break up biofilms by encoding depolymerases (enzymes) that degrade polysaccharides and penetrate biofilms [15].

A study showed that depolymerases produced by staphylococcal phage had the highest efficiency in clearing the biofilm [97]. Biofilms are dense with persister cells, non-dividing inactive cells, and the important causes of chronic and recurrent infection in humans [83]. Persister cells are those which reflect the failure of antibiotics to clear the bacterial population. They cause recurrent infection resulting in a high mortality rate [2]. The principle of phage behavior on persister cells has been the subject of interest in recent studies.

A report by Harper et al. showed that phages act on persister cells, in which phage remains dormant within persister cells. Phage lysis starts once persister cells revert to normal, which shows that phage therapy can be used in recurrent infections and is a promising alternative therapy for many incurable infectious diseases [37]. The implant-associated biofilm is caused by *Staphylococcus* spp., which remains to be a challenging endeavor to treat. To better understand the interaction between phage and the biofilm, it needs an in vivo study to put forth the outcome. Another study conducted by Seth et al*.* showed an adequate biofilm clearance in rabbit wound models infected with *S. aureus* wild-type UAMS1 strain and biofilm deficient UAMS929 strain [94, 95].

A study by Marion et al. showed the ability of phages to act against *S. mutans*, which significantly reduced the bacterial load. The reduction was recorded as 5 log CFU/ml in laboratory broth, 3 log CFU/ml in artificial saliva. It decolonized the *S. mutans* biofilm from the teeth surface within 24 h post-administration. The metabolic activity of phage-treated biofilm cells of *S. mutans* assessed by XTT assay (2,3-bis-(2- methoxy 4-nitro-5-sulfophenyl)-2H-tetrazolium- 5-carboxanilide salt) proved that the cell's metabolic activity remained stable even after 48 h, showing prolonged phage activity [102]. The oral biofilm comprises multi-species that live in symbiosis [34, 36], including a primary colonizer like *S. mutans*, *E. faecalis*, and later colonized by *Actinomyces* spp. Most of the species of oral biofilm show a distinct resistance to antibiotics. Despite the resistant nature, most of the oral microbiome inhabiting the root canals remains hidden from the human immune systems. *Enterococcus faecalis* is one of the most challenging bacterial species among the oral microbes; their ability to grow in high alkaline pH and thrive under glucose starvation has aided them to become a prominent flora in oral infections [65, 67]. As eradication of enterococcal biofilm is challenging, the application of phages has shown anti-biofilm properties in many studies. Another distinct nature of the *Enterococcus* is the ability to form recurrent infection, and their persistence in adverse conditions adds up the extremity of disease [11]. The severity of biofilm increases with bacterial age due to the persister cells. The phage application in mature biofilms is a breakthrough for phage therapy. A few reports have shown the ability of the phages to kill the mature biofilms as seen in *Enterococcus* phage, which cleared a 2-week-old biofilm in the tooth model within 24 h, thus showing a significance of phage therapy in biofilm clearance [42, 44]. The growing interest in developing synthetic phages for medical applications is an added advantage. A study conducted by Justine et al. has shown the significant impact of the engineered phage (EF11/ØFL1C (∆ 36)^PniSA^) on biofilm formed by both vancomycin-sensitive and vancomycin-resistant *E. faecalis* with 10–100-fold reduction of biomass post-phage treatment [102].

The research on bacteriophages infecting Gram-positive bacteria is very minimal. Just 3% of the complete genome of the *S. pyogenes* phage is currently available in the public database [34, 36]. The high frequency of generalized transduction makes the majority of the *S. pyogenes* phage temperate. For example, genomic analysis of the first lytic phage A25 isolated against *S. pyogenes* in the 1950s revealed an escaped lytic phage from prophage. Further investigation revealed homology to cluster in prophage of *S. pyogenes* M2, M3, and M4 strains [65, 67]. Several publications have also mentioned *S. pyogenes* phage's potential to lose integrase function, resulting in lysogenic-lytic shifts [66, 68]. Future research should focus on genetically engineered phages against *S. pyogenes*.

### Phage therapy against spore-forming bacteria

*Bacillus* is well known for infecting humans and causing gastrointestinal and pneumonia-like infections. *Bacillus anthracis* and *Bacillus cereus* are two clinically significant pathogens. *Bacillus* phage research started in the early 1950s, and their diversity is very high. *Bacillus* phages TP-13, TP-10, TP-18, and CP-51 were isolated from the soil and were all transduced phages. The majority of *Bacillus* phages have a limited host range, but there are exceptions [29, 31]. Several cases of *B. anthracis* treatment with phage therapy had been reported as being successful. The first phage study in *B. anthracis* was in the Thomas strain, and the bacteriophage was isolated from malignant pustules. In this experiment, mice were infected with 10^6^ CFU/mL of bacteria and treated with 10^9^–10^10^ PFU/mL of phage, resulting in 100% survival. More evidence exists that phage-encoded enzymes can treat *Bacillus* infections [21, 80, 91–93]. The biggest challenge in the treatment of *Bacillus* spp. is their ability to form spores. *An experimental analysis* by Henry et al. has shown that the phages lysis the spores. The study showed that high phage titers are required for the clearance of spores, thus promising against *Bacillus* infections [36, 38].

Despite rising antibiotic resistance, bacteriophages are rarely in use to treat *Clostridium difficile* infection (CDI). Most phages against *C. difficile* are temperate. There is no evidence in the literature showing a single phage with lytic activity against *C. difficile* which is the most significant limiting factor for in vivo testing of bacteriophage for CDI [33, 35, 75, 77, 90, 91]. There are no reports of phage treatment against spore-forming *Clostridium difficile*. Despite this, there are many studies undertaken to treat CDI with temperate phages [30, 32, 76, 78]. Due to this drawback, researchers are focusing on using a phage-encoded endolysin [95, 96]. An in vivo study with temperate phage was conducted using 80 strains of *C. difficile* belonging to 21 ribotypes of central epidemic origin. They came up with an interesting result that phage cocktails with lysogenic phages could kill most of the epidemic strains within 36 h. Post-infection and prevented the appearance of phage-resistant colonies [76, 78]. The impact of phages on the other species, *C. perfringens* and *C. tetani,* is less studied. Most of the studied lytic phages are used in phage typing [84, 85, 88, 89].

### Tackling phage resistance

**Fig. 1:**
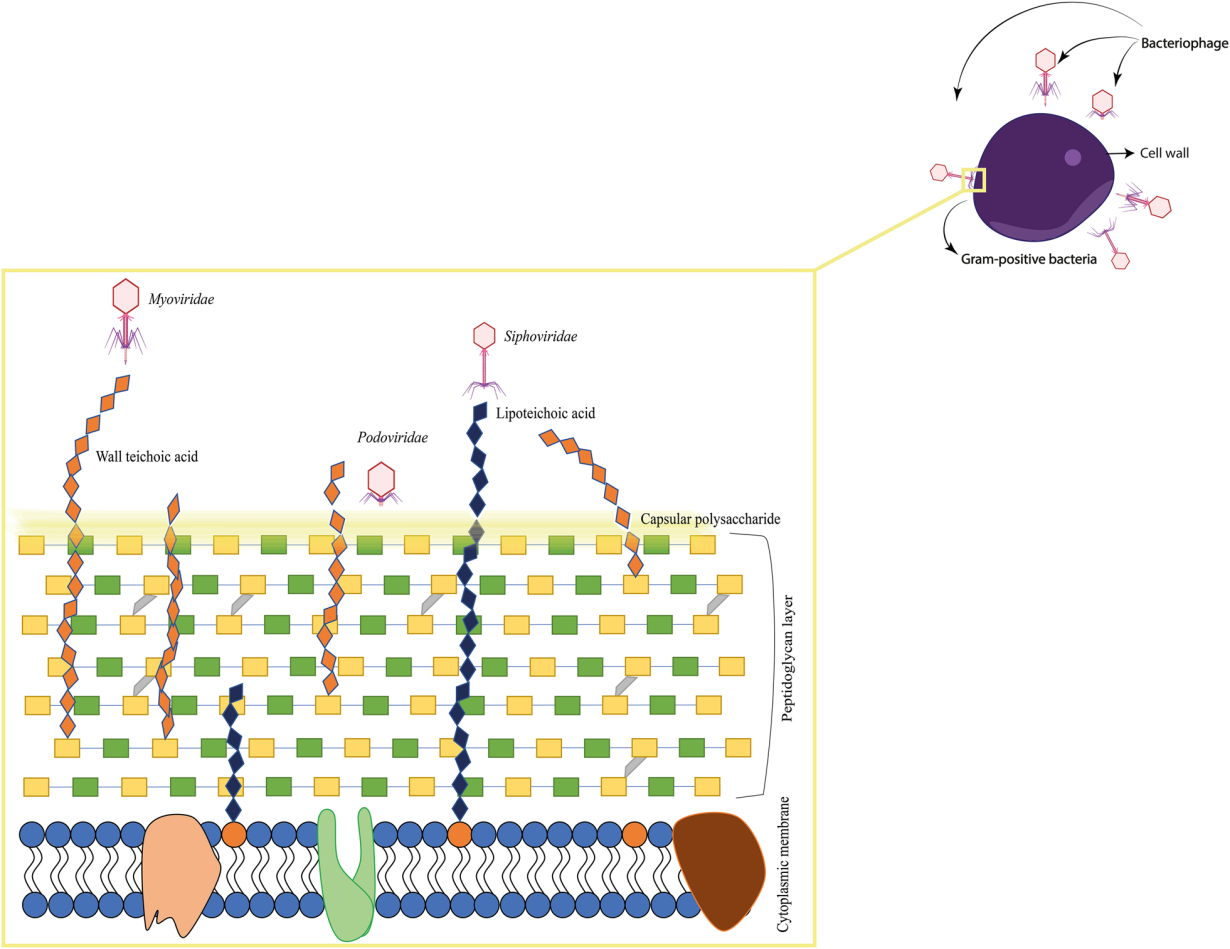
Diagrammatic representation of binding of bacteriophages to different receptors in the Gram-positive bacteria

Antibiotic resistance is an old phenomenon and revealed soon after the discovery of penicillin. Alexander Fleming has emphasized the risk of antibiotic resistance if bacteria are exposed to non-lethal levels of antibiotics. The concept of resistance is possible even for phages. So, phages should be used as an emergency or backup medication. In the present situation, the phages are used as interventional medicine when antibiotics fail to cure a bacterial infection. Luria and Delbrück [56, 58] reported the first case of rising phage-resistant mutants, and they observed the initial phage clearance followed by bacterial regrowth. Numerous researches are going on to deal with phage-resistant mutants, and phage cocktails are advantageous. Phage resistance may occur due to the mutation or shedding of the receptor/mutation to the receptor, CRISPR/Cas, restriction modifications, and selective pressure during evolution [9, 12, 23, 97]. The chance of growing competitive binding among the phages might influence the therapeutical index, which can overcome by using various combinations of phages [16]. By mechanism, the bacterial receptors determine the phage strain specificity. Few phages tend to use more than one receptor for their absorption [48, 50]. In Gram-positive bacteria, the outer layer is made up of carbohydrate moieties and teichoic acid, which are the central receptors of Gram-positive phages. The phage and bacterial receptor interaction in Gram-positive bacteria is represented in Fig. 1.

Phage cocktails can overcome the rising phage-resistant mutants where multiple phages are used against a target bacterium [32, 34]. However, the clinical evidence for the phage-resistant mutants is less recorded. Few studies have added that repeated dosage of phage for a stipulated period can eliminate the rising bacterial resistance [47, 49]. Phage cocktails can restrict the frequency of appearance of phage resistance and are formulated from huge phage stocks. Another aspect of phages as the therapeutic drug is challenging the bacteria with a synergistic combination of phage and antibiotics [55, 57]. It is an effective treatment strategy against bacterial infections, significantly eliminates the overuse of phages and antibiotics. It has been shown to hold a high therapeutic efficacy in which the antibiotics act as adjuvants to enhance the activity of phages by weakening the bacterial resistance mechanisms [20]. The study conducted by Sandeep et al. found that the administration of antibiotics before phage therapy improved the efficiency of staphylococcal phage by enhancing the phage adsorption and, thereby, increases the therapeutic efficacy. They claim that the synergistic effect might be due to antibiotic stress resulting in impaired protein production in bacteria [41, 43]. Another study showed clearance of MRSA biofilm when treated with phage–antibiotic combination (gentamycin, oxacillin, ciprofloxacin, linezolid) [22, 24]. A study led by Chan et al. showed that the bacteriophage receptor is related to the efflux system such as MexXY and MexAB conferred antibiotic susceptibility after phage treatment, as alterations in the efflux pump rendered the bacteria susceptible to the antibiotic [17]. A study conducted by Kirby et al. [44, 46] showed that treatment of *S. aureus* with phages combined with gentamicin reduced the rate of phage resistance in *S. aureus*. Many studies that showed the significance of synergistic activity of phage and antibiotic also showed that phage–antibiotic treatment might have some therapeutic complications because synergistic activity can vary between different strains of bacteria, antibiotics, and phage itself. Besides, there is a lack of proof to show whether the sub-inhibitory antibiotic regimen can interfere with the bacteriophage activity. However, we understand that there is limited information to prove the therapeutic efficacy of phage–antibiotic synergy [28, 30].

### Clinical trials of phages as therapeutic drug

Since the bacteriophage discovery, there have been numerous uncontrolled clinical trials to ascertain the therapeutic efficiency and safety of phage therapy for human use. Most chemotherapeutic agents used for treatment tend to exhibit side effects, while phage therapy has minor side effects but is less studied [40, 42]. The lack of controlled and randomized human trials makes it difficult to determine their actual side effects. Though there is a lack of proper clinical trials, the data from Georgia, Poland, and the former Soviet Union have a 90–95% success rate. The request to determine phage efficacy on humans has brought about many in vivo studies and clinical trials. The clinical outcome and the therapeutical efficacy of phage therapy are evaluated based on the frequency of phage requirement, route of administration, persistence of phages at the infection site, and anti-phage antibody production. The study conducted by Miedzybrodzki et al. at Ludwik Hirszfeld Institute of Immunology and Experimental Therapy, Wroclaw, Poland, found that the frequency of phage requirement for the treatment of infection caused by *S. aureus* was high (51.6% dosage frequency) with a low recovery rate of 36.7%. But the treatment of *E. faecalis* infection required low-frequency phage (11.1% dosage requirement) produced a 64.7% recovery rate, which showed that the therapeutical efficacy of the phage is directly proportional to the recovery rate [68, 70]. The route of phage administration also determines the efficiency of the treatment. Many studies have also shown that the route of administration has an indirect impact on the immune response, oral and topical applications had the lowest immune responses. Another factor that may have a partial impact on clinical outcomes is phage persistence at the site of infection. To test the persistence of phage at the site of infection, Eric et al. [72, 74] assessed the sinus sample taken after 24 h post-phage administration and found a minimal level of phages still circulating in the infected area. Another study led by Catherine et al. has shown that the presence of phage activity for the maximum of 25 days after the phage administration at the sinus region explains self-replicative and non-immunological phage clearance at the site of injection [67, 69]. Intraperitoneal injection of OMRII phage appeared in blood shortly post-administration in mouse bacteremia model, thus showing the importance of the route of parental administration based on the pharmacokinetics and the type of infection [5].

There are minimal notable case studies and clinical trials of bacteriophages against Gram-positive bacteria [56, 54, 106]. Among all, *S. aureus* bacteriophage has a well-documented clinical trial. Most of the whole phage and phage-encoded endolysins against *Staphylococcus* spp. are in phase II and phase III clinical trials. For instance, lysin exebacase (CF-301) produced by ContraFect, USA, is in phase III clinical trial, which acts against MRSA for treating endocarditis and prosthetic joint infections. GangaGen, India, has a phase II clinical trial of recombinant protein (ectolysin, P128) that kills MRSA. Most preliminary clinical trials are concerned about safety, while the efficacy and the clinical outcome of phage therapy are yet to be determined [39, 37, 106]. For example, the first FDA-approved phase I safety clinical trial conducted by Intralytix, USA, in chronic venous leg ulcer patients against *P. aeruginosa*, *S. aureus,* and *E. coli* has shown the safety in the administration of phages for wound ulcers [86, 87].

Recently, Centre Hospitalier Universitaire de Nīmes has registered for phase I/II clinical trial in collaboration with Pherecydes pharma to study phage (PHAGOPIED) against MRSA and MSSA in diabetic foot ulcer patients, which will complete in 2022. To our knowledge, there is no record of successful phase III clinical trial. But we strongly believe that phase III clinical trials in humans will help promote the globalization of phage therapy.

### Hurdles in making phage therapy a reality

Phage therapy is being used as a reserve drug in various parts of the world. When antibiotics fail, the use of phage therapy has become a usual treatment in most Western countries. The two most significant barriers to the globalization of phage therapy are (a) formulation and (b) commercialization. The question of 'What conditions must be met before phage therapy is administered to humans?' remains unanswered for the time being. Even though a few successful cases have occurred globally, not much is known about the immunological response. The lack of evidence and complete understanding of phage biology and its effectiveness has made medical practitioners less interested in phage therapy. Asking for an alternative is often less common among the patients due to lack of awareness and medical emergencies. Many developed countries have a modern medical system, in which medical insurance is one of the top-most priorities. Because phage therapy is not covered by standard medical insurance, it poses a financial challenge for many patients who cannot cover the costs [16]. Countries like Switzerland have planned for reimbursing such alternative medicines [45, 47], and few countries have started to approve them under medical claims. In order to bring phage therapy to a global extension, there requires a public awareness.

The inability to patent a phage product is one of the concerns for pharmaceutical companies to invest in phage production. Despite this, several companies are producing phages on a large scale for human use, and the global distribution of phage pharmaceutical products is considerably less. The phage centers are not globally distributed but confined within Europe, the USA, and Russia, which questions the therapy costs and travel expenses incurred during the treatment, putting an additional burden on patients from other nations. The global distribution of bacterial strains based on geographical locations makes the standard phage preparations more complicated. Thus, the challenge of developing a phage formula at the worldwide standard is another hurdle for phage therapy. The interaction between a bacteria and phage brings several factors into play, such as target bacteria, the metabolic state of the host, phage defenses, etc. Another major complication in constructing a universal phage formulation is the relationship between the bacteria, phage, and the human immune system [16].

**Fig. 2:**
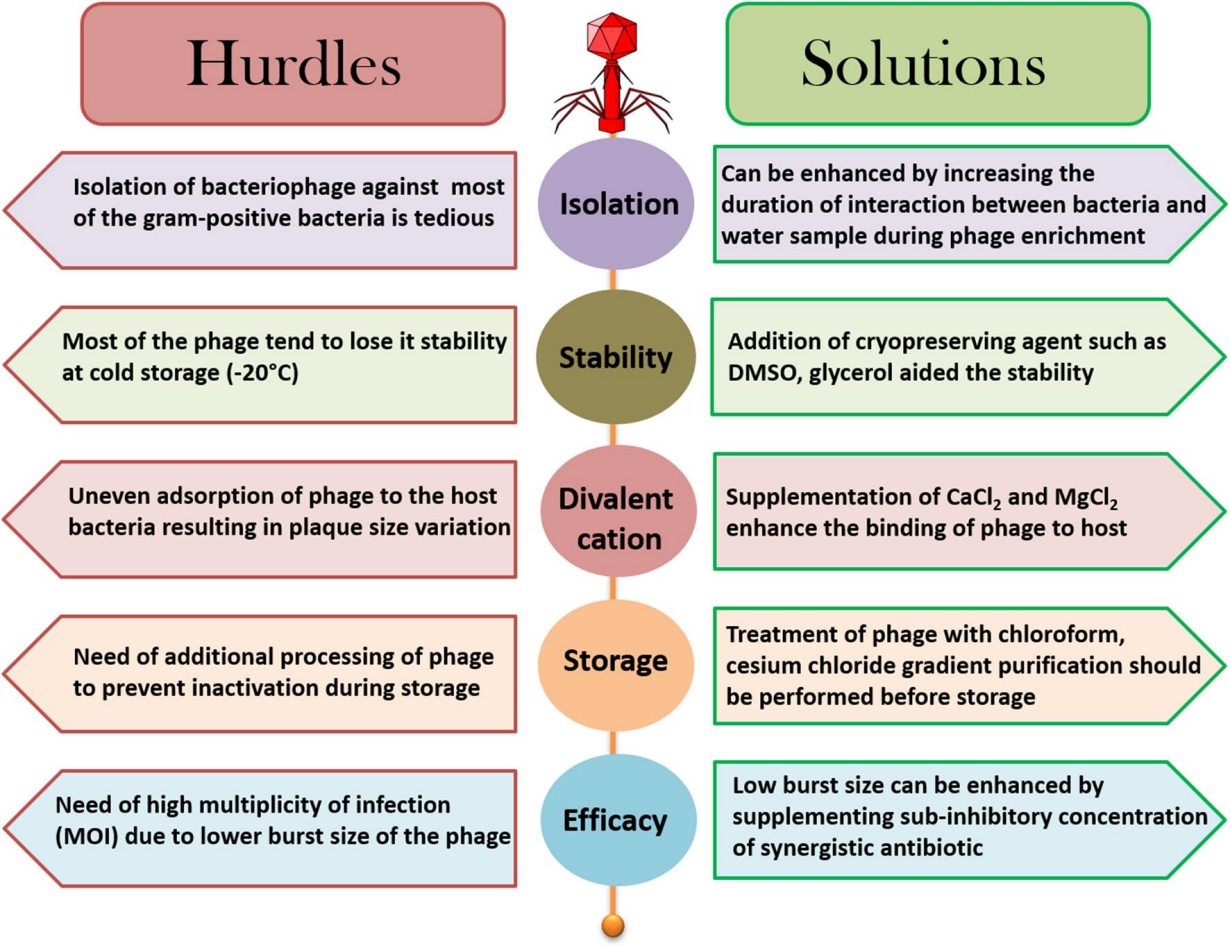
Schematic representation of the hurdles faced in phage laboratories and their potential solutions during the isolation of bacteriophages against Gram-positive bacteria

The phage therapy is not generalized globally because it varies from antibiotics in various aspects such as administration, formulation, and therapeutical index. One of the most challenging aspects is to establish broader host specificity. Naturally occurring genetic drifts can lead to resistant mutants that can be eliminated only when phage cocktails are used [78, 80]. There are numerous reports on the evolution of bacterial resistance to bacteriophage-based on different mechanisms (explained previously). The ready-to-make phage cocktails offer flexibility in therapy for encountering resistant bacteria, thereby reducing the need for universal phage formulations [17, 68, 70]. Alternatively, phage–antibiotic combinations are explored against resistant bacteria. Mechanically, a selective pressure-induced resistance may be reversed by exposing them to different antibiotic combinations. The documented results showed that the choice of antibiotics in phage synergy influences phage production. This enlightened the phage researchers to study the impact of phage–antibiotic synergy in treating antibiotic-resistant bacterial infections. The challenge for phage standardization does not start with commercialization; it emerges in laboratory experiments. A perfect outline to study phage and phage biology is critically essential before going for therapy. The possible challenges faced in studying Gram-positive phages during laboratory experiments are briefed in Fig. 2. Many factors differ between in vitro and in vivo* phage activity*, but molding phages for human use is one of the biggest challenges for the future.

### The market scope for global phage therapy

The commercialization of phage products is one of the biggest challenges faced by phage scientists. To bring a therapeutic product for human use, it has to pass through definite requisite which depends on the formulation, therapeutical efficiency, and the clinical (trials) outcomes. Phage therapy is a well-accepted treatment in the pre-antibiotic era. Still, it has not received global attention from the regulatory authorities to get approval for human use in the post-antibiotic era.

Commercialization of chemotherapeutical agents was easy as it tends to have fixed property, while phage therapy might need additional evidence on the efficacy, treatment strategy, and product development. In the past, Poland, Russia, and Belgium conducted clinical investigations under their declaration standards, and the export of phage products was less. Most of the countries are not coming forward to follow these declaration standards. Now phage commercialization should be of global demand, and the requirements should meet the international standards at the highest priority for the phage companies [50, 52, 64, 66]. There are few oldest phage centers to use phage products, (A) L’Oreal in France was the first to promote the phage products before the discovery and the development of antibiotics, and (B) Eli Lilly Co. sold their phage products in the 1940s. In those days, early clinical trials of bacteriophages were not accepted in the USA and other European countries, but countries like the former Soviet Union, Georgia, and Poland sold phage products for human use under their declarations [93, 94]. Eliava phage center in Georgia is profound in producing phages against Gram-positive bacteria such as *S. aureus*, *Enterococcus* spp. and *Streptococcus* spp*.* The biggest asset for the Eliava phage therapy center is the secret standard methods that are followed for years (https://eliavaphagetherapy.com/). The use of phage cocktails at different combinations is the success behind the phage center, where no reports of phage resistance up-to-date. Indeed, they keep an update about the phage host range with the current pathogenic strains, and if needed, they are replaced [50, 52].

Today, several companies have emerged to combat this war against antibiotic-resistant bacteria. To date, 51 companies are working on the various aspects of phages and phage products. Most companies are midway through their global clinical outputs, while a few are already in production and the last stage of their testing. The details of companies working on phage research against Gram-positive bacteria and their strategies for phage commercialization are outlined in Table 1. Companies develop a unique model to bring the phages into the market. For example, Adaptive phage therapeutics, USA, works on personalized phage therapy, while Locus bioscience, USA, works on synthetic phage production by utilizing CRISPR/Cas mechanism. The production/synthesis of phages using synthetic biology is of interest among the phage companies due to their ability to have a proprietary against the product [102]. Armata pharmacy, USA, produces phages against ESKAPE pathogens, creates a platform for synthetic phage production and increases its pharmacological importance. They are currently in the early stages (Investigational New Drug) of phage testing.

**Table 1:** Summary of phage pharmaceutical and their phage-based product

Name of the pharmaceuticals/centers	Location	Target Gram-positive pathogen	Name of the phage product	Platform	Product approval agent	Objective
Pherecydes Pharma	France	*Staphylococcus aureus*	PHOSA	Phage cocktail	N/A	To treat infections of bone and joint or diabetic foot ulcer infections
Armata Pharmaceuticals	USA	*Staphylococcus aureus, Streptococcus mutans*	AP-SA02	Synthetic phages	FDA	They use synthetic phages in which phage are engineered to express C16G2 antimicrobial peptide for treating tooth decays caused by *S. aureus* and *S. mutans*
Eliava Phage Institute	Georgia	*Staphylococcus aureus, Staphylococcus epidermidis, Streptococcus pyogenes, Streptococcus sanguis, Streptococcus salivarius, Enterococcus* spp.	Staphylococcal, SES, FERSISI, INTESTI, PYO Bacteriophages	Natural phages and personalized phage cocktails	N/A	Largest phage therapy center that works on various number of infections caused by resistant pathogens
Intralytix Pharmaceuticals	USA	*Staphylococcus aureus*	PhagoBioDerm	Natural phage	FDA	Targeting the leg ulcers caused by *Staphylococcus aureus*, they have successful completed the phase I clinical trials
Adaptive Phage Therapeutics	USA	*Staphylococcus aureus*	N/A	Personalized phage cocktails	Not approved by FDA, currently they are treating patient on eIND	The host range of the phages (deposited in phage bank) is determined by calorimetric method against the pathogen isolated from the patients and given in the cocktail for therapy
Locus Biosciences	USA	*Clostridium difficile*	crPhage™	CRISPR/Cas engineered phage	FDA	To treat recurrent *Clostridium difficile* infection (CDI)
Phagelux	China	*Staphylococcus aureus,Staphylococcus epidermidis*	PL-01-SS,PL-03-BM,PL-06-FC,PL-01-SZ,	Natural phages, endolysin	N/A	For treatment of infection caused by *Staphylococcus spp.* such as burn wounds, ulcers, acne, eczema and implant related. They are in the pre-clinical studies of the phage products
Phagomed	Austria	*Staphylococcus aureus*	N/A	Natural phage cocktails	Austrian Research Promotion Agency (FFG)	To treatment biofilms and implant associated infections
Microgen	Russia	*Staphylococcus aureus*, *Streptococcus* spp.	Sextaphag®, Complex pyobacteriophage, Streptococcus bacteriophage	Natural phage cocktails	N/A	To treat various number of infections caused by *S. aureus and Streptococcus spp.* in combination with other phages of Gram-negative pathogens
GangaGen	India	*Staphylococcus aureus*	P128 (ectolysin)	Phage-encoded endolysin	N/A	They are in the Phase II clinical trial of lysin against MRSA
ContraFect	USA	*Staphylococcus aureus* *Streptococcus pneumonia* *Enterococcus faecalis* Group B *streptococcus* *Bacillus anthracis*	CF-301 (Exebacase), G.P.lysins	Phage-encoded endolysin	FDA	To treat endocarditis, prosthetic joint infections caused by MRSA and also against other Gram-positive bacterial infections. Lysin is used in combination with SOC antibiotic (e.g., vancomycin, daptomycin) that is found to enhance the efficacy. Exebacase is the first lysin to enter phase III clinical trial in USA

*N/A* information not available, *FDA* Food and Drug Administration, *eIND* emergency investigational new drug

To date, very few phage products get FDA approval to conduct clinical testing. According to Data Bridge Market research among pharma companies in 2021, the peak market growth in global phage therapy is expected to occur between 2021 and 2028. North America (USA), Europe (Germany, France), Asia–Pacific (India, Japan, and China), the Middle East, and Africa will be the most promising countries in the expanding phage market (https://www.databridgemarketresearch.com/reports/global-bacteriophages-therapy-market). As the bacteriophages are (bacterial) viruses, there is always a concern among the public about their safety. Therefore, creating public awareness about the safety of phage therapy is indispensable. Similarly, significant commercialization depends on the cost that should be appealing from the customers’ perspective to take phages from the laboratory to the patient's bedside.

## Discussion

Phage therapy is the best complimentary from nature in the post-antibiotic era. Although detailed clinical trials are required, phage therapy will find a niche as an antibacterial drug in modern medicine. Phage therapy research is advancing faster than expected; still, a lot needs to be studied from a therapeutic aspect. Although we have a record of ‘phage therapy centers’ treating patients for years, the globalization of phage therapy is in its infancy. The acceptance of phages as a therapeutic drug will be the first success in product globalization. There is a significant amount of phage population in the humans known as phageome. Therefore, the introduction of phages as a therapeutic agent is considered safe. The future of phage therapy will largely be determined by the research output (clinical trials), the cost per treatment, and the production [51, 53].

Many Gram-positive bacterial infections remain untreatable, including MRSA and VRE. Despite the availability of last-resort antibiotics, the mortality rate is still high for these MDR bacterial infections. Treating such infections using phage therapy has to be overlooked by physicians, funding bodies, governments, and policymakers. The use of a combination of phage–antibiotics is a growing trend in phage research, but their implementation is hampered by a lack of proper execution and authorization. Though the number of ongoing clinical trials is limited and very few clinical trials are in phase II, there is plenty of information from Georgia and Poland that shows that phages are effective therapeutic agents [104].

The recombinant technology (rDNA) in phage biology has brought out a lot of improvement in phage preparation for a therapeutic cure [83, 84]. The information recovered from the whole genome sequence has unlocked numerous information on phage biology [46, 48, 85, 86]. The ideas and outputs of pharmaceuticals and research centers have shown that phage therapy is an effective alternative. The use of phage therapy in secondary bacterial infections such as in COVID-19, influenza virus outbreak, and cancer is an unexplored topic in phage therapy [13, 43, 45, 103]. A strategic plan to tackle such infections using phage therapy needs investigation.

The lack of acceptance of phage therapy is due to the lack of standard clinical trials in humans and proper regulatory guidelines. The knowledge gap in phage biology is mainly due to the lack of funds or investments to carry out research endeavors. Other barriers include age-dependent treatment strategies, improper clinical guidance, and large-scale phage production to meet a global market [25, 27]. Despite all hurdles, the efforts to bring phage therapy as an alternative to antibiotics are growing to meet the global standards.

## Conclusions

A survey by WHO has shown that growing antibiotic resistance is life-threatening in which even a minor injury would lead to complications. The recent pandemic of COVID-19 has shown us the consequence of not having a prior medication/vaccine, which leads to fatalities globally. Antibiotic resistance is also growing to be a global healthcare crisis; acting early would save the days left in the post-antibiotic era. Growing technical advancements such as next-generation sequencing and high-throughput screening have opened a vast space for research in evaluating the efficacy of phage interactions in the humans and increased the chances of genome analysis and speeding-up the characterization of phages. Many research and clinical trials carried out in different parts of the world have shed light on phage therapy as an effective alternative. Though phages will not replace antibiotics, they will be the best compliment that will revolutionize the era of non-antibiotics.

## Data Availability

The dataset analyzed in the study are available in the GenBank database and Clinicaltrials.gov. Other data studies are presented in the form of figures and tables.

## Abbreviations

IND: Investigation new drug
FDA: Food and Drug Administration
NIH: National Institute of Health
WHO: World Health Organization
MRSA: Methicillin-resistant *Staphylococcus aureus*
EPS: Exopolysaccharide
CDI: *Clostridium difficile* Infection
UTI: Urinary tract infection
USA: United States of America
VRE: Vancomycin-resistant enterococcus
MDR: Multi-drug resistant
rDNA: Recombinant DNA
